# Preserving and Using Germplasm and Dissociated Embryonic Cells for Conserving Caribbean and Pacific Coral

**DOI:** 10.1371/journal.pone.0033354

**Published:** 2012-03-08

**Authors:** Mary Hagedorn, Virginia Carter, Kelly Martorana, Malia K. Paresa, Jason Acker, Iliana B. Baums, Eric Borneman, Michael Brittsan, Michael Byers, Michael Henley, Michael Laterveer, Jo-Ann Leong, Megan McCarthy, Stuart Meyers, Brian D. Nelson, Dirk Petersen, Terrence Tiersch, Rafael Cuevas Uribe, Erik Woods, David Wildt

**Affiliations:** 1 Department of Reproductive Sciences, Smithsonian Conservation Biology Institute, Front Royal, Virginia, United States of America; 2 Hawaii Institute of Marine Biology, University of Hawaii, Kaneohe, Hawaii, United States of America; 3 Laboratory Medicine and Pathology, University of Alberta, Edmonton, Alberta Canada; 4 Department of Biology, The Pennsylvania State University, University Park, Pennsylvania, United States of America; 5 Department of Biology and Biochemistry, University of Houston, Houston, Texas, United States of America; 6 Columbus Zoo and Aquarium, Powell, Ohio, United States of America; 7 General BioTechnology, Indianapolis, Indiana, United States of America; 8 Invertebrate Exhibit, Smithsonian National Zoological Park, Washington, District of Colombia, United States of America; 9 Rotterdam Zoo, Rotterdam, The Netherlands; 10 Department of Anatomy, Physiology and Cell Biology, University of California Davis, Davis, California, United States of America; 11 Fishes Department, New England Aquarium, Central Wharf, Boston, Massachusetts, United States of America; 12 Aquaculture Research Station, Louisiana State University Agricultural Center, Baton Rouge, Louisiana, United States of America; Leibniz Center for Tropical Marine Ecology, Germany

## Abstract

Coral reefs are experiencing unprecedented degradation due to human activities, and protecting specific reef habitats may not stop this decline, because the most serious threats are global (i.e., climate change), not local. However, e*x situ* preservation practices can provide safeguards for coral reef conservation. Specifically, modern advances in cryobiology and genome banking could secure existing species and genetic diversity until genotypes can be introduced into rehabilitated habitats. We assessed the feasibility of recovering viable sperm and embryonic cells post-thaw from two coral species, *Acropora palmata* and *Fungia scutaria* that have diffferent evolutionary histories, ecological niches and reproductive strategies. *In vitro* fertilization (IVF) of conspecific eggs using fresh (control) spermatozoa revealed high levels of fertilization (>90% in *A. palmata*; >84% in *F. scutaria; P*>0.05) that were unaffected by tested sperm concentrations. A solution of 10% dimethyl sulfoxide (DMSO) at cooling rates of 20 to 30°C/min most successfully cryopreserved both *A. palmata* and *F. scutaria* spermatozoa and allowed producing developing larvae *in vitro*. IVF success under these conditions was 65% in *A. palmata* and 53% in *F. scutaria* on particular nights; however, on subsequent nights, the same process resulted in little or no IVF success. Thus, the window for optimal freezing of high quality spermatozoa was short (∼5 h for one night each spawning cycle). Additionally, cryopreserved *F. scutaria* embryonic cells had∼50% post-thaw viability as measured by intact membranes. Thus, despite some differences between species, coral spermatozoa and embryonic cells are viable after low temperature (−196°C) storage, preservation and thawing. Based on these results, we have begun systematically banking coral spermatozoa and embryonic cells on a large-scale as a support approach for preserving existing bio- and genetic diversity found in reef systems.

## Introduction

Coral reefs are living, complex ecosystems. While occupying a global spatial footprint about the size of Bolivia, coral reefs still have high diversity (>800 species recognized in the stony corals) [1] and provide invaluable ecosystem services to the planet – as nurseries for marine fish and invertebrates, natural storm barriers for coastlines and for food and pharmaceuticals used by humans. As a group, corals are evolutionarily ancient [2], but recently coral reefs have been experiencing unprecedented degradations. Locally, reefs are damaged by pollution, nutrients and sedimentation from poor land-use, fishing and mining practices [3]. Globally, increased levels of greenhouse gases are warming and acidifying oceans, which is making corals more susceptible to stress, bleaching and newly emerging diseases [4], [5], [6]. The coupling of climate change and anthropogenic stressors has caused a widespread reef crisis [4], [5], [6], [7], [8], [9].

The status of Caribbean reefs is dire, with elkhorn *(Acropora palmata*) and staghorn *(Acropora cervicornis*) corals, once the foundation species for this region, showing widespread population declines [10], [11], [12]. For example, recent surveys have revealed that 80 to 99% of these populations have been extirpated [13], [14], leading to the dubious distinction of being the first of this group to be listed as threatened under the Endangered Species Act [15], [16], [17].

In general, corals can reproduce sexually (via planktonic or floating larvae) or asexually (by fragmentation and resettlement). The former can generate genetic diversity, whereas the latter maintains the status quo of the species. Caribbean *A. palmata* and *A. cervicornis* appear to most commonly propagate via asexual breakage and then re-attachment of branches, in contrast to recruitment of sexually-produced larvae [18], [19], [20]. When sexual reproduction does occur, larvae are produced *once* annually when hermaphroditic colonies release eggs and sperm (spawning) and fertilization occurs. Because (1) occurrence and spawn time can be unpredictable in Caribbean Acroporids and (2) *A. palmata* and *A. cervicornis* do not generally self-fertilize [21], reproductive success can be sporadic and encumbered in the isolated, small populations common in the Caribbean. In certain areas in the Florida Keys, genotypic diversity can be so low (e.g., only one clone per reef) that sexual reproduction is all but impossible [20]. Such reproductive failures contribute to the continuing decline of Caribbean Acroporids [22], [23]. Additionally, young recruits are being adversely affected by increasing macroalgal cover on reefs [24], [25]. Together, these factors increase the likelihood of elkhorn and staghorn coral extinctions within the next 5 to 20 years [26], [27].

Historically, *ex situ* populations of wildlife species have been important as ambassadors for public awareness (i.e., zoos and aquaria), and can serve as a hedge against extinction, to generate species-specific biological knowledge impossible to collect *in situ* and for producing offspring for reintroduction into native habitats [28]. Some of the best practices from *ex situ* wildlife management programs have application to conserving coral. First, advances in coral husbandry now allow for maintenance of *ex situ* populations. The coral conservation consortium of aquarists and scientists, called SECORE (SExual COral REproduction, www.secore.org), successfully collected *A. palmata* egg-sperm bundles, used *in vitro* fertilization (IVF) and reared and transported larvae so that there are now >3,000 young adults in public zoos and aquaria around the world [29], [30]. Second, preliminary findings suggest that biomedical reproductive methods can be applied in corals [31]. Similar to many wildlife groups [28], assisted reproductive technologies could be relevant to propagating wild coral populations. Especially advantageous is the field of cryobiology – low temperature biology – and understanding the mechanisms that allow the successful cooling, freezing and thawing of germplasm or embryos. Because cells that are frozen and banked properly can retain viability for years (or even centuries) without DNA damage, this is a means to safeguard all existing species and gene diversity [32], [33], [34]. More specifically, these ‘genome resource banks’ offer (1) large samples of preserved and protected gene pools that can be used to ‘seed’ shrinking populations, including those with heterozygosity, or re-populate depauperate habitats, (2) easy and inexpensive transport of genetic materials among living populations, (3) extended generation intervals and (4) vast improvement of access to biomaterials for scholarly research [35], [36], [37]. We believe these benefits also have relevance to coral conservation, including applying a suite of tools related to *ex situ* husbandry and cryomethodologies.

Our studies focus on the *in vitro* reproduction and cryobiology of two coral species, *Acropora palmata* and *Fungia scutaria*. The goal was to examine commonalities in reproductive biology and cryophysiology that might lead to a singular, effective cryopreservation procedure broadly applicable to diverse coral species. Our comparative approach was important because our target study species differ in mode of sexual reproduction (hermaphroditic versus gonochoristic or separation of the sexes in different individuals) and occupy different ecological niches (reef-building coral versus isolated). *A. palmata* is a large colonial species occupying only shallow water, with hermaphroditic polyps producing positively buoyant egg/sperm bundles that develop into free-swimming larvae [1]. In contrast, *F. scutaria* is a solitary, small-sized species that lives in a variety of reef zones as gonochoric individuals that produce negatively buoyant gametes. In an earlier study, we offered preliminary evidence that coral spermatozoa can survive cryopreservation [31] based largely on post-thaw motility. However, to date, the functional viability of such cells for fertilization has not been shown. The objectives of this investigation were to examine (1) the *in vitro* reproductive characteristics and the influence of cryoprotectants, chilling and freezing on the motility and fertilization capacity of *A. palmata* and *F. scutaria* spermatozoa for creating a successful cryopreservation protocol, (2) the cryosensitivity and post-thaw viability of dissociated embryonic cells from 12 h *F. scutaria* and (3) the feasibility of large-scale, field banking to begin preserving coral bio- and genetic diversity.

## Methods

Due to limited gamete availability from naturally brief spawning durations, collection periods for both species were short. *A. palmata* studies were conducted in Puerto Rico over 1 wk (∼4 nights), whereas *F. scutaria* investigations were done in Hawaii over 4 mo (∼16 nights). Although we report the findings made during a single annual breeding cycle for both species, 4 years of preliminary studies were performed to establish our field and laboratory practices.

In terms of general methods, we studied two species with different types of reproduction. *A. palmata* produced egg/sperm bundles which were collected from several specimens at once and then separated into eggs and sperm. Spermatozoa were pooled, counted, diluted and checked for motility, and then pooled sperm was subjected to various cryopreservation testing for toxicity and sensitivity to freezing. Each sample, then was, thawed and used to inseminate fresh, conspecific eggs *in vitro* on that same night. For *F. scutaria*, sperm and eggs were expelled from different male and female animals, so were collected separately. However, all subsequent processing and evaluation procedures were the same as with *A. palmata*.

### Gamete collection

**Figure 1:**
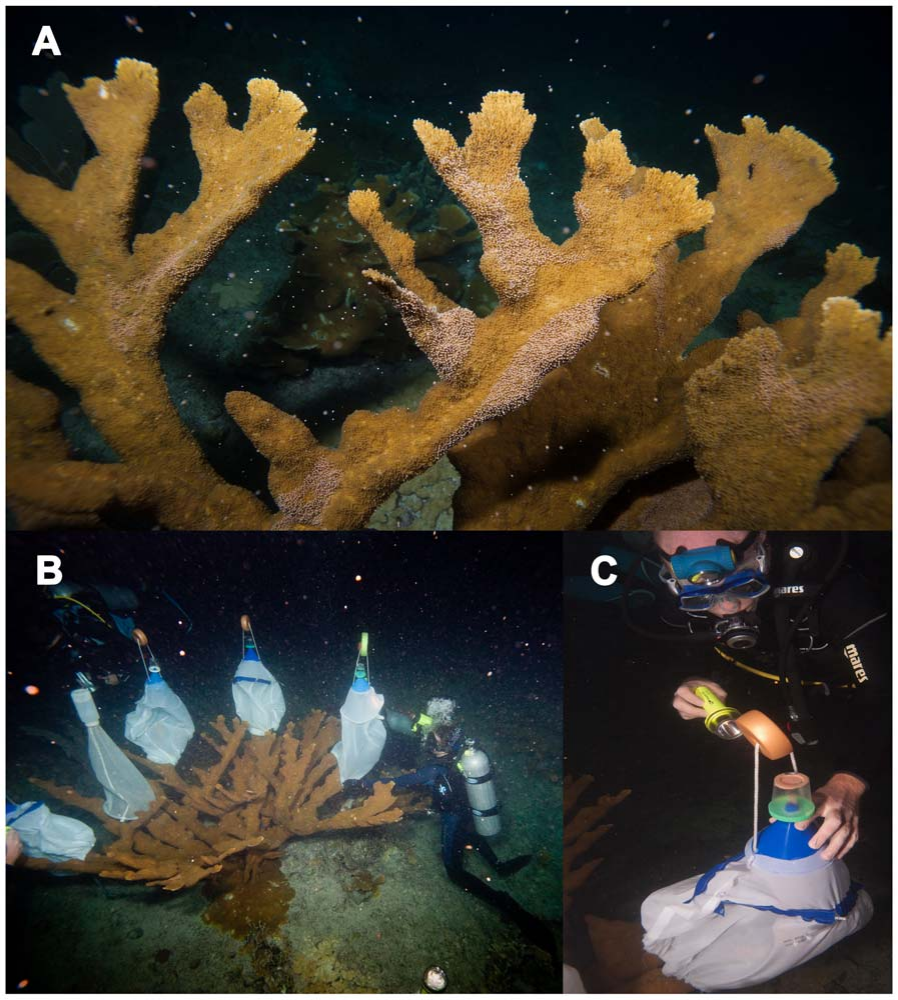
Scientific SECORE divers placing several collection nets on a large *A. palmata* colony prior to spawning in the evening. A) *A. palmata* setting, note the pink egg/sperm bundles resting on the surface of the brown colony prior to release into the water column. B) Scientific divers placing the specially-designed nets on a colony to collect egg/sperm bundles. C) Egg/sperm bundles were collected in the cup at the top of the net. Photos contributed courtesy of the Pittsburgh Zoo & PPG Aquarium, Photo ©2010 Paul A. Selvaggio.

Recovery of coral reproductive cells was performed with the appropriate permits from the Departments of Land and Natural Resources in both Puerto Rico and the State of Hawaii. *A. palmata* eggs and sperm were collected during the annual spawn from Trés Palmas Reserve (Rincón, Puerto Rico) in August, 2009. Egg-sperm bundles were collected with specially designed fine mesh nets made from polyester silk (Silkessence, Jo Ann's Fabrics www.joann.com, Fig. 1). These nets were pillow-shaped and approximately 1 m long and wide with a nylon synch cord at the bottom and a 10 cm-wide funnel hot-glued to the top of the 10 cm tapered net. Straddling the funnel tip was a 100 ml urine cup lid with a 2.5 cm hole glued in place. This allowed urine cups to be attached and removed during the collection process. The net was held buoyant by a small float (8 cm in diameter, 2.5 cm thick) so the urine cup remained parallel to the water surface, allowing the buoyant bundles to float upwards through the inverted funnel into the 100 ml urine cup. Thirty to 45 min before the anticipated onset of spawning (∼2100 hr), divers cinched the nets to a coral branch (typically at a depth of 1 to 5 m) on a given colony. These nets were placed only on colonies that were ‘setting’, meaning bundles were visible on the coral's surface. After ∼45 min of release of egg/sperm bundles, collection cups were removed from the stationary lid while held inverted underwater to keep the bundles in place, recapped with a spare lid and then transferred to shore within 30 min. For the *A. palmata* study, egg-sperm bundles were transferred to 50 ml conical tubes to reduce the volume of water, and the eggs and sperm were separated by gentle agitation of the tubes. The eggs floated in the tube, whereas sperm were suspended throughout. Concentrated spermatozoa were removed to a separate 50 ml plastic tube and maintained in the original salt water until cell concentration was determined. In parallel, eggs were serially washed (at least 3×, or until the rinse water was clear and the eggs floated and moved unencumbered at the top of the tube) with 50 ml rinses of 0.2 µm-filtered seawater (FSW) at room temperature (typically 28 to 30°C). Unless stated otherwise, all other described solutions were made using 0.2 µm- FSW (1,000 mOsm) generated by passing raw seawater through a Nalgene Steri-cup filtration system.

Fungia scutaria adults were collected from various shallow reef flats in Kaneohe Bay and then maintained in shallow running seawater tables at the Hawaii Institute for Marine Biology, University of Hawaii. We adhered to the gamete collection and larvae rearing methods of Krupp [38] and Schwarz et al. [39]. Briefly, *F. scutaria* spawns over 2 to 3 nights for multiple months each summer (June to September), ∼2 nights after a full moon [38]. Two hr before spawning onset (∼1800 hr), adults were placed into individual 2 L plastic bowls filled with seawater, and individual disposable pipettes (Fisher Scientific) were placed in each bowl to minimize cross contamination of gametes between bowls. On the basis of observing a uniform milky cloud from the mouth of individual coral specimens, spermatozoa were immediately aspirated using a plastic, disposable pipette and held in individual 50 ml conical plastic tubes. By contrast, eggs released from the mouth and visualized as a particulate white cloud were collected from the bottom of the bowl using a disposable pipette. There was no opportunity for gamete interaction, and all eggs were immediately washed three times in ∼100 ml FSW and then placed into a glass beaker to count and dilute at a temperature of 23 to 26°C for IVF. Meanwhile, spermatozoa were maintained at the same temperature in raw seawater (1,000 mOsm) for later processing. Generally, gametes were cleaned and separated as rapidly as possible, and always <1 hr post-recovery. Sperm remained concentrated in FSW until density was determined, whereas eggs remained in FSW until placed in vials for reproductive assessments. Two teams worked in parallel, one processing, evaluating and cryopreserving spermatozoa with the other separating eggs.

### Assessments of sperm concentration and motility

Sperm motility estimates for each collection were made immediately by placing a ∼10 µl aliquot onto a glass slide, adding a cover-slip (only for *A. palmata*) and visualizing the sample via phase optics (200×) on a Zeiss Student or Olympus BX41 microscope. Specifically, a qualitative quartile method was used with the slide moved to assess a minimum of three full frames. The collective fields then were estimated as having sperm expressing <25, 50, 75 or >90% progressive or forward motility. The process was repeated with two additional 10 µl samples, and an overall motility average calculated. Sperm concentration in each diluted sample was determined using a standard hemocytometer method [40]. For both species, spermatozoa were pooled from at least three males (most commonly 5 to 7 donors) on a given collection night, usually into a total volume of 25 to 50 ml. This strategy also increased total sperm available for each cryopreservation trial, but also was considered as a useful future approach for ensuring heterozygosity in thawed inseminates (especially important in *A. palmata* where there are barriers to self-fertilization [21]).

### Assessment of cell viability

Viability of thawed embryonic cells was defined as those cells with intact cell membranes. We used a standard propidium iodide (Invitrogen) assay that exposed cells to a fluorescent dye that intercalates with DNA nucleotides and is generally excluded by intact cells [41]. Stained cells were analyzed by flow cytometry (Accuri C6, Accuri Cytometers, Inc. Ann Arbor, MI USA), and 10,000 events were analyzed per sample. This instrument measured total number of cells in a sample, and propidium iodide revealed the number of damaged cells. In brief, coral cells were exposed to a 2.4 mM stock solution of propidium iodide (1 µl stock solution/250 µl of cells) and incubated for 10 min at 24°C. Control (dead) samples were created by subjecting cells to three cycles of freezing to −196°C and thawing at 30°C (without cryoprotectant) before staining with propidium iodide and flow cytometry analysis. Data were evaluated using Accuri software (CFlow Plus, Ver. 1.0.202.1).

### Experiment I: Sperm viability and IVF validation

This experiment determined the optimum concentration of fresh spermatozoa needed to achieve fertilization *in vitro*. This was essential because all subsequent cryosensitivity experiments evaluated sperm survival post-treatment based on the capacity of these cells to interact with and fertilize eggs from the same species in culture [21]. For both species and all trials, IVF was conducted in 20 ml scintillation vials (Thermo Fisher Scientific), each containing 5 ml of FSW, 30 to 50 eggs and no (control) or fresh spermatozoa of varying concentrations. For *A. palmata*, eggs were exposed to spermatozoa for 5 min and then serially rinsed three times in 5 ml of FSW or not rinsed at all, with fertilization success scored 12 hr later (see below). For *F. scutaria*, eggs were exposed to sperm for 12 hr according to Krupp [38] and Schwarz et al. [39]. For *A. palmata*, we examined a shorter fertilization time (5 min), because we were concerned about bacterial contamination of a 12 hr long incubation from decaying, large, yolk-filled eggs and the high sperm concentrations.

**Figure 2:**
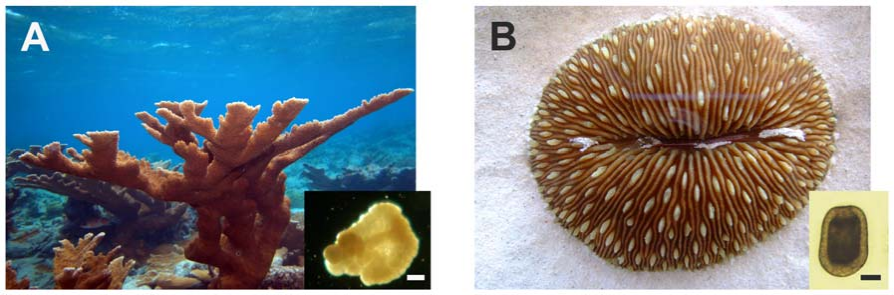
Adult and larval forms of *A. palmata* and *F. scutaria*. **A**) Adult and developing *A. palmata* larvae (inset) at the cornflake stage at ∼24 h. Scale bar = 50 µm. Adult photo by R. Williams, Smithsonian Institution. **B**) Adult and developing *F. scutaria* larvae at the swimming stage. Scale bar = 50 µm. Embryos that reached these stages were scored as successfully developed.

Initially and for validation purposes, we determined optimal concentration of fresh spermatozoa needed to initiate fertilization (defined below). Table 1 lists the treatments tested for *A. palmata* and *F. scutaria* in these initial trials. After adding spermatozoa (at the test concentration), the original vials were maintained at 27 to 29°C (without agitation) and developmental progression assayed at 12 hr post-insemination. Fertilization was based on numbers of developing larvae (versus un-inseminated eggs) quantified under a Wild dissecting microscope (50×). More specifically, for *A. palmata*, we calculated the percentage of larvae that developed to the ‘cornflake’ or ‘prawn-chip’ stage by12 hr of culture at 27 to 29°C, described as fertilized by Okubo and Motokawa [42] (Fig. 2A). For *F. scutaria*, we determined fertilization as the proportion of larvae developing to a motile state by 12 hr at 27°C (Fig. 2B) according to Krupp [38] and Schwarz et al. [39].

**Table 1:** Experiment I: Fresh sperm treatments for *in vitro* fertilization.

Species	No. individual egg donors*	No. pooled sperm donors	Fresh sperm concentrations tested
*A. palmata*	8	0	None added (control)
*F. scutaria*	8	3 to 7**	10^6^, 10^7^, 10^8^ cells/ml
*F. scutaria*	16	0	None added (control)
*F. scutaria*	16	7 to 10	10^5^, 10^6^ cells/ml

* Thirty to 50 fresh eggs were used per pooled sperm sample.

** Eight replicates per female for each sperm concentration.

### Experiment II: Cryoprotectant toxicity

The purpose of this study was to identify candidate cryoprotectants by examining the effect of cryoprotectant toxicity on the ability of fresh sperm to fertilize eggs from *A. palmata* and *F. scutaria.* All cryoprotectant solutions described in this paper were prepared originally at double-strength concentration (vol/vol) and diluted 1∶1 with 0.2 µm FSW to produce the final test concentration. This eventually facilitated our fieldwork as this approach allowed quick mixing of one-part cryoprotectant and one-part seawater with cells to achieve our final target concentration. Spermatozoa were exposed to each cryoprotectant (protocols and gamete donors are summarized in Table 2 for each cryoprotectant trial) for 20 min at 27 to 29°C (for *A. palmata*) and at 23 to 26°C (for *F. scutaria*) and then assessed in the IVF assay. While various cryoprotectants and concentrations were tested per species (Table 2), ultimately we selected a total of four treatments for subsequent studies: 5 and 10% dimethyl sulfoxide (DMSO) and propylene glycol (PG).

**Table 2:** Experiment II: *In vitro* fertilization after exposing fresh sperm to various cryoprotectant treatments.

Species	No. individual egg donors*	No. pooled sperm donors	Treatment
			*1. Preliminary Screening Experiments*
*A. palmata*	4	3 to 7***	10 separate test cryoprotectants: 5 and 10% dimethyl sulfoxide (DMSO)^1^, 5 and 10% propylene glycol (PG)^1^,5% glycerol^1^, 5% methanol^1^, 5% ethylene glycol^1^, 5% methylene glycol^1^, 5% 2-methyl-2,4-pentanediol^2^, 5% 1-methoxy-2 propanol^2^
*F. scutaria*	*–*	*–*	
			*2. Tests With Candidate Cryoprotectants*
*A. palmata*	4	3 to 7***	4 separate tests cryoprotectants: 5 and 10% dimethyl sulfoxide (DMSO)^1^, 5 and 10% propylene glycol (PG)^1^,
*F. scutaria*	10	3 to 7***	4 separate test cryoprotectants: 5 and 10% dimethyl sulfoxide (DMSO)^1^, 5 and 10% propylene glycol (PG)^1^,

1 Sigma-Aldrich, St Louis, MO, USA.

2 Acros Organics, Fair Lawn, NJ, USA.

* Thirty to 50 fresh eggs were used per pooled sperm sample.

*** An inseminate concentration of 10^6^ sperm/ml was used for each species.

### Experiment III: Sperm and embryonic cell freezing sensitivity

This experiment used the collective findings of Experiments I and II to assess and apply cryopreservation methods for cells from both species. There were four components: 1) determining optimal cooling/freezing rate for *A. palmata* and *F. scutaria* spermatozoa; 2) evaluating the impact of freeze-thawing spermatozoa from both species; 3) examining the efficacy of cryopreserving dissociated *F. scutaria* embryonic cells; and 4) implementing systematic cryobanking of spermatozoa (*A. palmata*) and embryonic cells (*F. scutaria*).

Identifying an optimal cooling rate for cryopreservation, or rate at which cells are frozen, is often an iterative process due to the wide range of potentially perturbing factors (e.g., natural species differences, volume of the sample, packaging, type and concentration of cryoprotectant, among others). For this reason, most cryo-studies begin by examining the influence of cooling rate as well as cryoprotectant type and concentration. Therefore, we first conducted a preliminary trial of *A. palmata* spermatozoa using 10% DMSO versus PG, two common cryoprotectants used in marine cryobiology studies [43]. Early findings (data not shown) demonstrated that both of these cryoprotectants at this concentration resulted in post-thaw motility after sperm were cooled/frozen at a rate of 20 to 30°C/min. This information was used as the foundation for developing the more complicated comparative studies reported below.

Specifically, for each species we compared two concentrations of DMSO or PG (5% versus 10%) and the original cooling rate of 20 to 30°C/min with a slower rate of 8 to 15°C/min (*A. palmata*, n = 3–7 pooled sperm donors/cooling range and n = 28 egg donors; *F. scutaria*, n = 3–7 pooled sperm donors/cooling range and n = 16 egg donors). To achieve the faster cooling rate, a liquid nitrogen (LN_2_) field apparatus was used (described below), while the slower cooling rate was achieved using an electronic, controlled-rate freezer (for *A. palmata*, a Grant Asymptote EF600; for *F. scutaria*, a Planar Freezer Kryo 360). When either the LN_2_ field box or the controlled-rate freezer was used, the sample temperature was lowered from 25 to 29°C to −80°C at the specific cooling rate, then plunged into LN_2_ for at least 10 min before thawing. Motility and viability of the *F. scutaria* sperm treated with 5 and 10% DMSO and PG also were assessed with phase microscopy and flow cytometry (see above) before and after thawing (n = 10 females, n = 3–7 pooled sperm donors each of 3 nights).

It is challenging to cryopreserve living biomaterials in the field, so robust methods that suited coral cells were developed. Our simple field freezing apparatus consisted of a Styrofoam box (27.5 cm long×22.5 cm wide×20.5 cm high) partially filled with LN_2_ fitted with a lid (to maintain the vapor) that allowed 20 cryovials to float ∼1 cm above the LN_2_ vapor. The float consisted of a frame of floating polyresin ‘flip-flop material’ (Ben Franklin Craft Store) with the frame opening covered with a rectangle of aluminum mesh. Two sections of aluminum cryocanes (Thermo Fisher) bent into a v-shape were snuggly attached to the mesh.

With this apparatus, a typical freezing run consisted of exposing spermatozoa to either 5 or 10% DMSO or PG and then cooling at 20 to 30°C/min. For each species, we evaluated pooled sperm samples from three to seven sperm donors on each collection night. Each pooled sample was evaluated for initial cellular motility and sperm count (as described above), pooled and then diluted in FSW to 2×10^9^ cells/ml (*A. palmata*) or 2×10^8^ cells/ml (*F. scutaria*). A double-strength concentration of either 10 or 20% DMSO or PG was added to permit the process of equilibration to begin over 20 min at 27 to 29°C (*A. palmata*) or 23 to 26°C (*F. scutaria*). Each 1 ml sample then was pipetted into a 2 ml cryovial, affixed to the v-shaped holder that was quickly (<5 sec) placed onto the LN_2_ surface (3 to 5 cm below the top of the box) of the Styrofoam freezing box, ensuring ∼1 cm gap between the cryovial and the LN_2_ fraction. The lid was replaced, the samples held in the vapor for 5 to 6 min until the cryovials reached −80°C and then the whole apparatus plunged into LN_2_ (−196°C) for at least 10 min or longer before analysis or transfer to a dry shipper (for short term storage and transportation from the field). Preliminary testing with a thermocouple (Omega HH147 RS-232 Data Logger Thermometer, Stamford, CT) indicated that the 1 ml content within each cryovial was being exposed to a freezing rate of 20 to 30°C/min rate. To examine the slower cooling rate (8 to 15°C/min), cells were diluted with cryoprotectant, loaded into the 2 ml cryovials, placed into the control-rate freezer, cooled to −80°C, and then individual cryovials were plunged into LN_2_ (−196°C) for at least 10 min before analysis.

For both species, a subset of frozen aliquots was thawed the same day to evaluate the effect of cooling rate and cryoprotectants on IVF success as well as sperm motility and viability (only *F. scutaria*). This process involved removing the cryovials from LN_2_ which then were exposed to 30°C in a water bath for 2 min. A 50 µl aliquot of each sample was pipetted immediately into each of the vials containing 30 to 50 eggs so that the amount of viable sperm in the vial was ∼1×10^6^ cells/ml (while maintaining a low and negligible cryoprotectant concentration in the vial). An additional 100 µl of the thawed sperm sample was used to evaluate post-thaw motility and cellular viability (as described above for *F. scutaria*).

For embryonic cell cryopreservation, we examined the cryosensitivity of these cells, specifically those of *F. scutaria*, because only this species was in proximity to our laboratory in Hawaii that had the required equipment. Based on the sperm experiments, we tested DMSO and PG, both at a 5 and 10% concentration. Methods were modified from Falciatori et al. [44] who had studied mouse spermatogonial stem cell lines. Since there was a lack of information on coral stem cells or the optimal time to dissociate such embryos, we collected thousands of *F. scutaria* embryos at ∼12 h post-fertilization, a time when cells were beginning to differentiate into various lineages (Hagedorn, unpublished data). Embryos were dissociated simultaneously by placing into a 6% pronase (10 mg/ml) solution in FSW for 2 min followed by mechanical disruption by pressing through a cell-sorting basket with 40 µm mesh screen (Falcon, Fisher Scientific) using the rounded end of a plastic spatula. The resulting cell suspension (∼10^7^ cells/ml) was collected into 1 ml Eppendorf tubes and then vortexed to produce a consistent suspension that was confirmed by examining microscopically (400 to 800×) for uniform cellular dispersion. After allowing cell settlement for 30 to 60 sec, the pronase solution was gently aspirated and discarded. Sufficient FSW was added to the admixture to produce a final 500 µl volume that was supplemented with 150 µl of 10% bovine serum albumin in FSW. This solution was centrifuged (5,900×g, 5 min; Eppendorf Centrifuge 5415D) and the supernatant removed. A 500 µl aliquot of bovine serum albumin (4% in FSW) was added, the cell pellet re-suspended with a pipettor, and then the 500 µl volume transferred into a 2 ml cryovial. Then, 500 µl of a double-strength cryoprotectant was added to each cryovial (e.g., of 10 or 20% DMSO or PG) and mixed using a pipette. Eighteen vials were placed into the slots of a 4°C alcohol freezer (Mr. Frosty Freezers, Nalgene, Fisher Scientific) that was held for 20 min at 4°C (to equilibrate the cryoprotectant), and then the alcohol freezer was transferred into a −80°C freezer for at least 8 h to produce a cooling rate of 0.5°C/min. From the time of placing cryovials into the alcohol container through the −80°C freezer exposure, at least one vial was monitored with the Omega thermocouple to generate a temperature profile. After reaching −80°C, the vials were quenched in LN_2_ for at least 10 min to reach −196°C and then either stored long-term in a 35 L LN_2_ dewar or thawed in a 30°C water bath for 2 min and assessed for viability (as described above). Each of the four cryoprotectant treatments was evaluated simultaneously four times over the course of 3 different days.

Once the fundamental protocols were developed, we systematically banked spermatozoa from *A. palmata*, first by holding in a dry shipper (for transportation from the field) to permanent storage in LN_2_ dewars. A similar approach was used for embryonic cells of *F. scutaria* that were maintained in 35 L LN_2_ tanks in Hawaii.

### Data analysis

All statistical evaluations were performed using Graphpad Prism 5.0 (San Diego, CA) and Microsoft Excel (version 2007). Percentage data were arcsine-transformed, and significance was recognized at *P*<0.05 (for all tests). Results among groups were evaluated statistically by one-way analysis of variance (ANOVA), and an F value reported. For nonparametric assessments, a Kruskal-Wallis or a Mann Whitney test was used.

## Results

### Experiment I: Optimizing IVF for coral

**Figure 3:**
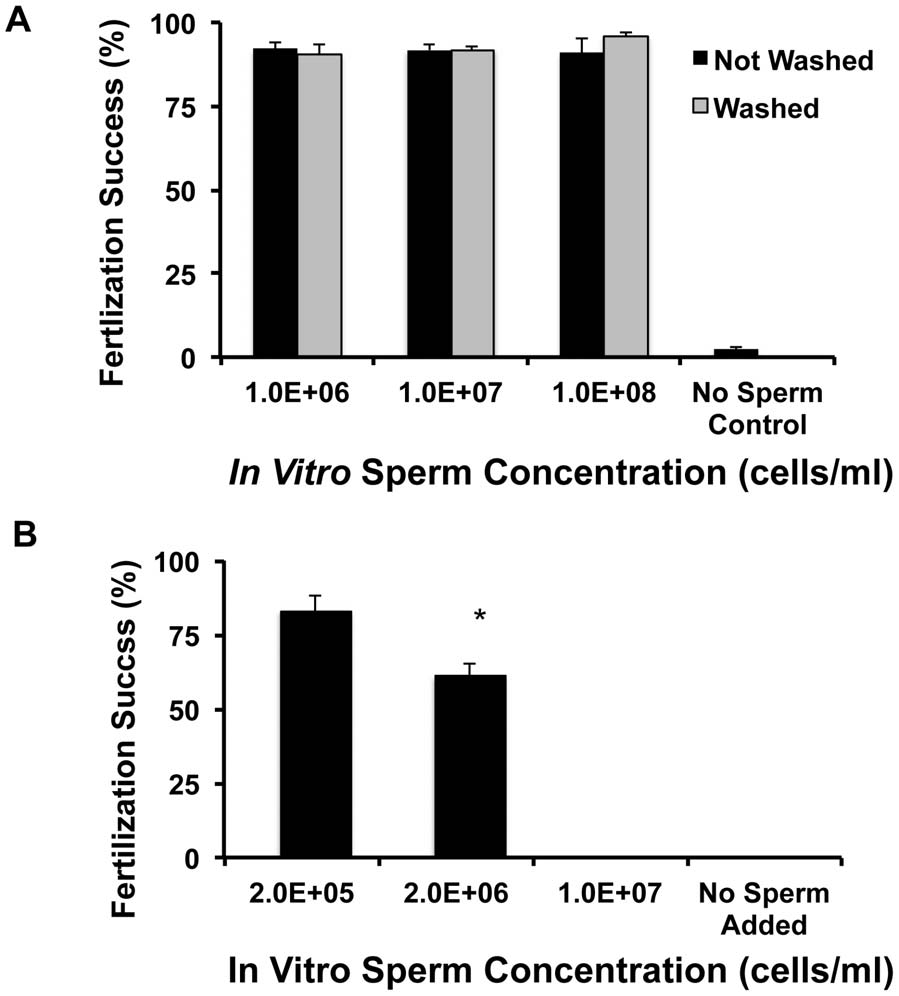
Species-specific sperm concentrations were not necessary for successful *in vitro* fertilization. **A**) Regardless of the *A. palmata* sperm concentration used (10^6^ to 10^8^ cells/ml), a successful *in vitro* fertilization success of >92% was observed regardless of whether the eggs were exposed to the sperm for 5 min (grey bars) or overnight (black bars) (*P*>0.05; ANOVA). **B**) Both sperm concentrations for *F. scutaria* produced uniform IVF results (*P*>0.05; Mann-Whitney).

Overall, fertilization rates *in vitro* were consistently high in both species using fresh spermatozoa, >85% IVF success for *A. palmata* and >75% for *F. scutaria*. There were no differences in fertilization success in either *A. palmata* or *F. scutaria* on the basis of fresh sperm inseminate concentration or duration of the insemination interval (for *A. palmata*). For *A. palmata*, >92% of eggs fertilized whether in the presence of 10^6^, 10^7^ or 10^8^ sperm cells/ml or when incubated from 5 min and then rinsed or 12 h and not rinsed (*P*>0.05, ANOVA, F = 70, N = 8 egg donors and 3 to 7 sperm donors, Fig. 3A). Due to being expelled directly into the water, it was not possible to produce a highly concentrated solution of *F. scutaria* spermatozoa. For this species, >75% of eggs fertilized whether in the presence of 10^5^ or 10^6^ sperm cells/ml *(P*>0.05, t-test, N = 16 egg donors and 7 to 10 sperm donors, Fig. 3B). The incidence of self- fertilization was negligible in both species (*A. palmata*, 2.0%; *F. scutaria*, 0%).

Although we dealt mostly with pooled samples, we did examine fresh motility prior to pooling. Individual male motility from both species varied considerably from 25% to >90% and appeared to be night-dependent during an individual spawning period. In *F. scutaria*, these individual variations did not affect the fertilization success of the pooled sperm samples (*P*>0.05, Kruskal-Wallace test, N = 16 egg donors and 7 to 10 sperm donors); insufficient data were available for individual *A. palmata* to evaluate statistically. Thus, for IVF there was no preferred sperm concentration for either species, and *A. palmata* eggs fertilized just as efficiently with a 5 min versus 12 h sperm exposure.

### Experiment II: Determining cryosensitivities

**Figure 4:**
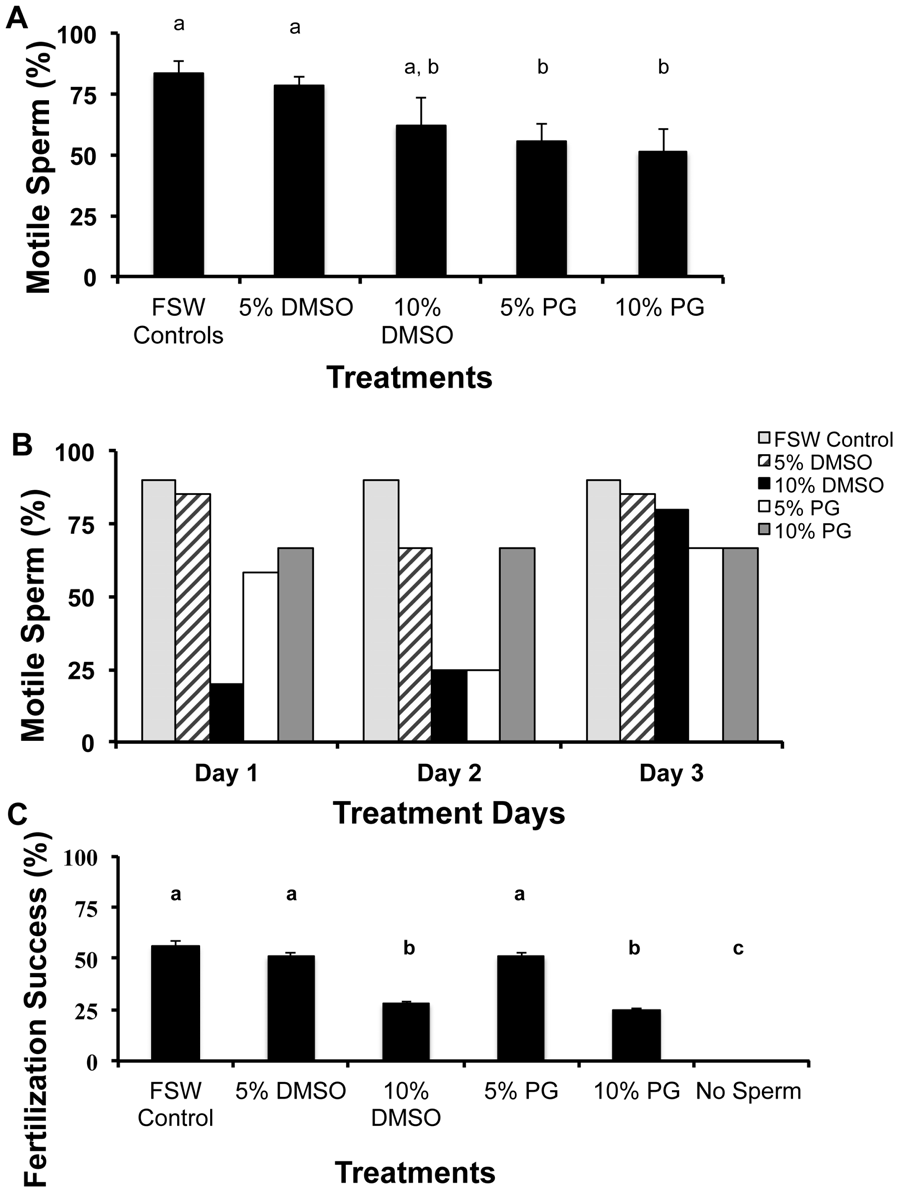
*F. scutaria* sperm were sensitive to cryoprotectants (no sperm exposed to freezing in any of these treatments). **A**) If the prefreeze motility data (N = 7) for several spawning periods were averaged across the test cryoprotectants, there was no clear indication which cryoprotectant solution might impact the motility the least, except DMSO solutions might be slightly preferable. For analysis, the % motility was measured in quartiles, which were converted into numbers from 1 (25% or less motile) to 4 (>90% motile). Bars with the same letters were not different (*P*>0.05; Kruskal-Wallis test), but bars with different letters were different (*P*<0.05; Kruskal-Wallis test). FSW controls included fresh sperm with no cryoprotectant. **B**) However, if the effect of the cryoprotectants on *F. scutaria* sperm motility for one individual spawning period in the month of July was examined each day, there was a variability pattern in sperm motility each night. Note on Day 1 and 2, the toxicity of 10% DMSO was high (low motility), whereas on Day 3 it was low (high motility). **C**) In contrast, 10% DMSO and PG solutions caused a 30 to 40% decrease in fertilization success for fresh *F. scutaria*, whereas the 5% solutions did not. Bars with the same letters were not different (*P*>0.05; ANOVA), whereas bars with different letters were different (*P*<0.05; ANOVA).

Fresh spermatozoa from *A. palmata* were not sensitive to cryoprotectant concentrations. Regardless of the 10 cryoprotectant exposure treatments tested (Table 2), cryoprotectant-exposed fresh sperm fertilized >85% of inseminated eggs at a rate no different from controls (93%) (*P*>0.05, Kruskal-Wallis test, N = 4 egg donors, 3 to 7 sperm donors). Because there was no difference among cryoprotectants and we wanted to develop a single, effective cryopreservation protocol for both species, all subsequent *A. palmata* experiments evaluated only 5 and 10% DMSO and PG solutions. By contrast, fresh *F. scutaria* spermatozoa were sensitive to cryoprotectant treatments with both sperm motility (*P*<0.05, ANOVA, F = 3.5, N = 10 egg donors and 3 to 7 sperm donors, Fig. 4A) and IVF success (*P*<0.05, ANOVA, F = 14.8, N = 10 egg donors and 3 to 7 sperm donors, Fig. 4C) adversely affected by higher (10%) compared to lower (5%) DMSO and PG concentrations. One of the most interesting aspects of *F. scutaria* sperm physiology was its variable response in motility to cryoprotectants from night-to-night within a spawning season (Fig. 4B), a variation that was obscured when data were averaged (Fig. 4A). For example, sperm unexposed to cryoprotectant had a 90% motility rating on each of the three spawning nights. By contrast, fresh sperm treated with 10% DMSO varied from 20 to 85% motility on different nights. *F. scutaria* also differed from *A. palmata* in that IVF success for the former was reduced by 30 to 40% compared to controls in the presence of 10% DMSO or 10% PG (*P*<0.05, ANOVA, F = 14.8, N = 10 egg donors and 3 to 7 sperm donors, Fig. 4C). The adverse influence was lost when the DMSO and PG concentrations was lowered to 5%. Thus, a higher concentration of either of these cryoprotectants had a toxic influence on IVF success of sperm from *F. scutaria* but not *A. palmata.*

### Experiment III: Producing a successful cryopreservation protocol

A singular, effective cryopreservation method for *A. palmata* and *F. scutaria* sperm and embryonic cell freezing was achieved using 10% DMSO at varying cooling rates.

**Figure 5:**
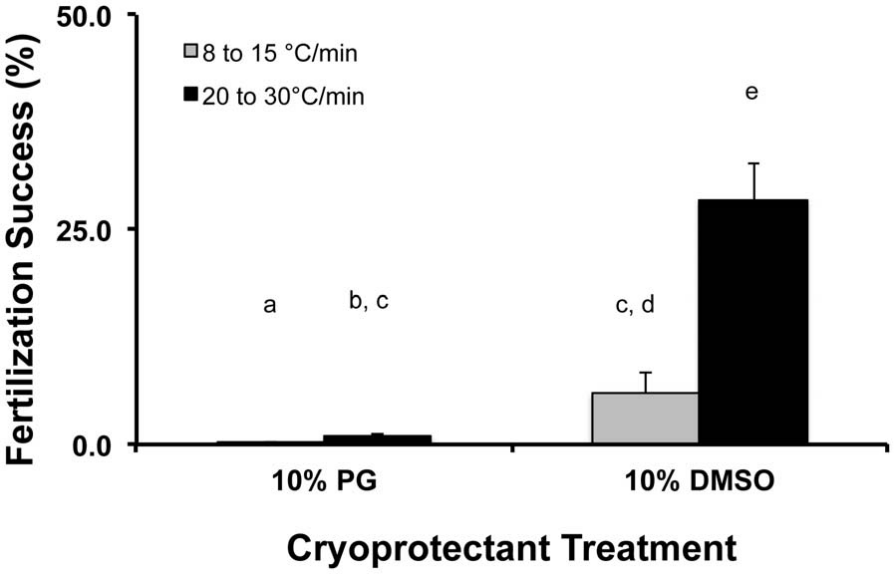
The effect of cooling rate for successful *F. scutaria* spermatozoa cryopreservation (no sperm exposed to freezing in any of these treatments). Two freezing ranges were examined and two cryoprotectant solutions (*F. scutaria*, n = 3–7 pooled sperm donors/cooling range and n = 16 egg donors) and then the influence of cooling rate on IVF success. Only the 10% DMSO at a cooling rate greater than 20°C produced reasonable post-thaw fertilization. Bars with the same letter were not different (*P*>0.05; ANOVA), whereas bars with different letters were different (*P*<0.05; ANOVA).

Preliminary cooling rate studies were conducted with *A. palmata*, suggesting that sperm cooled at 20 to 30°C/min in 10% DMSO had up to 75% post-thaw motility, whereas rates from 8 to 15°C/min using either 5 or 10% DMSO or PG resulted in no IVF success. For *F. scutaria*, a cooling rate of 20 to 30°C/min using 10% DMSO produced the highest IVF success (28%), whereas 8 to 15°C/min and the other solution reduced IVF success (6% or lower) (*P*<0.05, ANOVA, F = 49; N = 20 egg donors and 12 sperm donors, Fig. 5). Therefore, a cooling rate of 20 to 30°C/min was chosen for cryopreserving sperm from both species.

**Figure 6:**
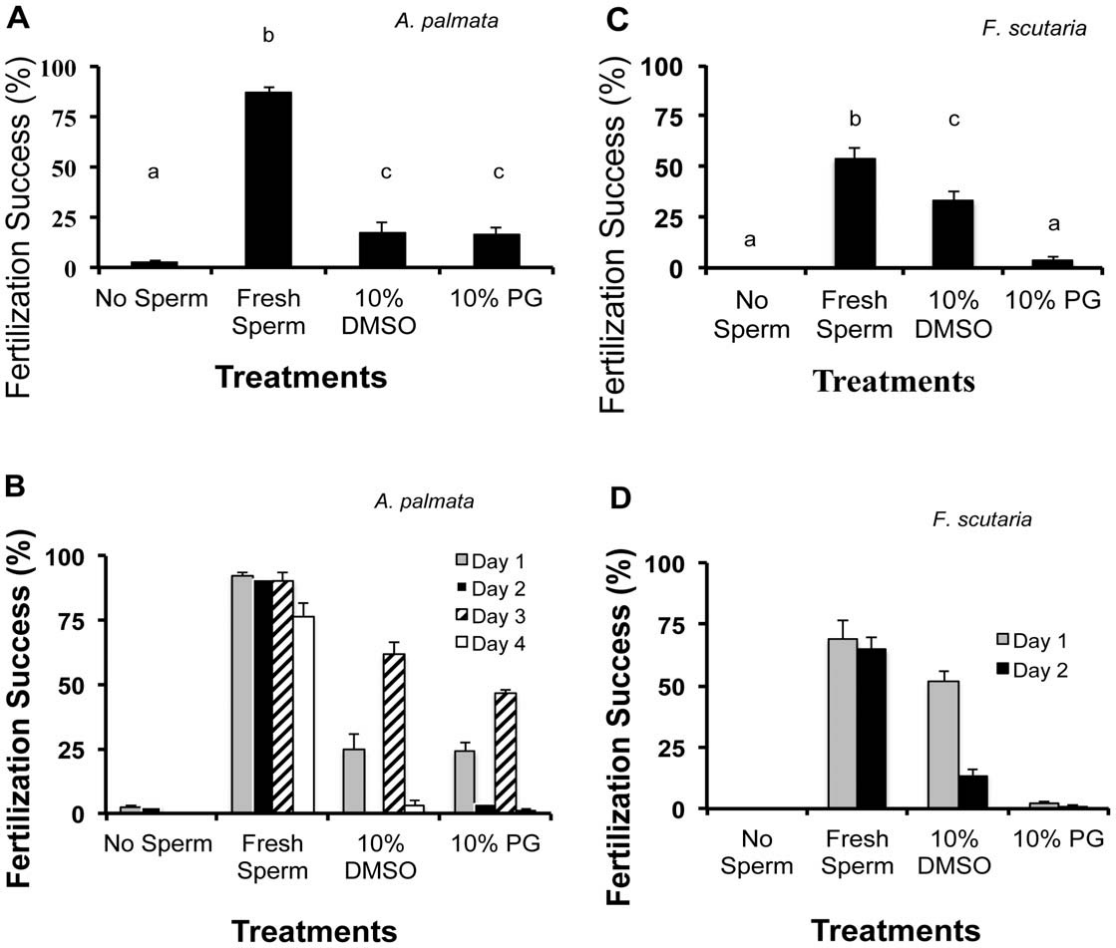
Cryopreservation of coral sperm (all sperm exposed to freezing in these treatments). **A**) *A. palmata* sperm were cryopreserved at cooling rates 20 to 30°C/min using 10% DMSO and PG, and IVF success was assessed and averaged over the single spawning period. This averaged graph revealed no difference between the two cryoprotectants and a mean fertilization success of ∼18%. **B**) However, if the *A. palmata* fertilization success during the spawning period was graphed by day, a 65% fertilization success occurred on Day 3 with 10% DMSO, whereas it was 25, 0 and 3% on Day 1, 2 and 4, respectively. For the first 3 nights, the control with fresh sperm held at ∼90%, then fell to 76% on the fourth evening. **C**) *F. scutaria* sperm were cryopreserved at rates 20 to 30°C/min using 10% DMSO versus PG, and fertilization success was assessed and averaged over two spawning periods (July and August 2010). Averaging indicated that 10% DMSO was the preferred cryoprotectant, and (as in *A. palmata*) there was no variability in time in terms of physiological responses during a spawning season. **D**) Variability in *F. scutaria* IVF success after cryopreservation over two nights of a single representative month (August 2010). Fresh sperm IVF success held steady at 65%, but sperm cryopreserved with 10% DMSO varied from 52 to 13% on the two evenings. Bars with the same letters were not different (*P*>0.05; ANOVA), whereas bars with different letters were different (*P*<0.05; ANOVA).

Once the freezing rate range was established, a more detailed examination of how the two cryoprotectants affected IVF success was undertaken. Freezing the sperm from these coral species always reduced IVF success compared to fresh counterparts (Fig. 6A, C). For *A. palmata*, either the 10% DMSO and 10% PG treatment produced similar IVF success (*P*>0.05, ANOVA, N = 16 egg donors and 14 to 20 sperm donors, F = 100.5; Fig. 6A); however, both reduced fertilization *in vitro* by ∼78% compared to fresh controls. By contrast, 10% DMSO was advantageous compared to PG for *F. scutaria* spermatozoa, the former reducing fertilization by ∼27% compared to fresh controls (*P*>0.05, ANOVA, N = 16 egg donors and 14 to 20 sperm donors, F = 40.2, Fig. 6C).

However, when the data were examined on a nightly basis, there was a night-of-sperm-collection effect on subsequent ability of spermatozoa from either species to survive cryopreservation (Fig. 6). On the basis of a nightly evaluation, 10% DMSO consistently produced the highest post-thaw IVF success in both species. Specifically for *A. palmata*, post-thaw fertilization success was 65% on the third night of spawning compared to 25% for counterparts collected on the first night, 0% on the second night and 3% on the fourth night (Fig. 6 B), all handled exactly the same way. A similar observation was made for 10% DMSO-frozen *F. scutaria* spermatozoa, with a fertilization success of 51% for the first night of collection compared to 13% for the second night (Fig. 6 D).

**Figure 7:**
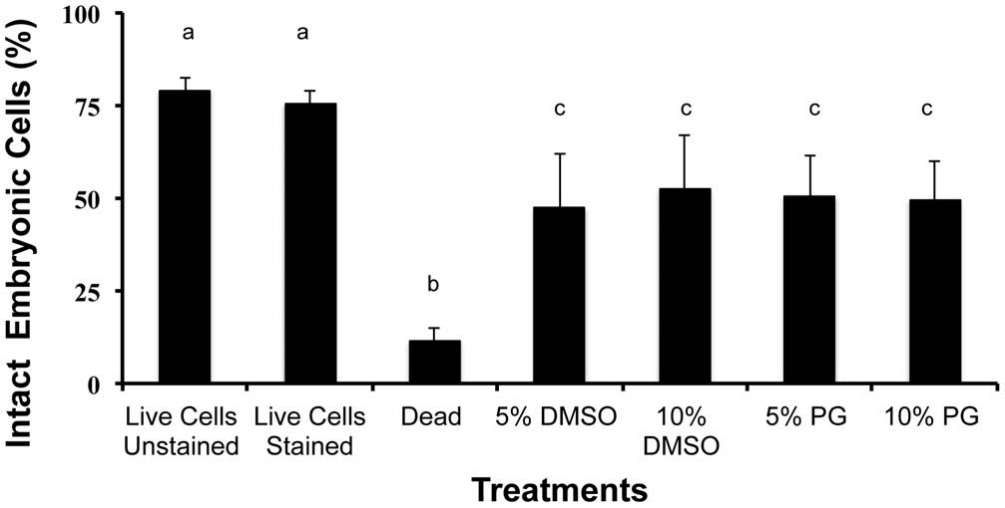
Embryonic *F. scutaria* cells after cryopreservation (all cells exposed to freezing in these treatments). Mean post-thaw viability of *F. scutaria* cells was ∼50% for all cryoprotectants tested. Ten thousand events were measured for each sample. Controls (the three left bars) were live-stained and unstained cells and 100% dead cells that produced control data for the flow cytometer (i.e., 100% intact versus 100% dead). Bars with the same letters were not different, whereas bars with different letters were different (P<0.05; Kruskal-Wallis test).

In terms of survival of *F. scutaria* embryonic cells, ∼50% of the dissociated cells were viable post-thawing, regardless of cryoprotectant type or concentration when the 0.5°C/min cooling rate was used (Fig. 7). Interestingly, the ability of these cells to withstand cold temperature also appeared dependent on night of collection. Within a coral-spawning interval of 3 days, the proportion of embryonic cells with intact membranes ranged from 50 to 80%.

Using the knowledge generated in our earlier experiments and our findings that post-thaw functionality is achievable with 10% DMSO and cooling rates of 20 to 30°C/min for sperm and 0.5°C/min for embryonic cells, we have proceeded with the first ever, systematic, large-scale banking of coral sperm and embryonic cells. Our focus largely has been on *A. palmata* spermatozoa due to the now threatened status of this species. This effort to date has resulted in the storage of >450 *A. palmata* sperm samples and >500 billion spermatozoa. The donor sites for these collections have included individuals from over 2 linear km of the Trés Palmas Reserve (Rincón, Puerto Rico). Frozen and labeled cryovials stored in LN_2_ were transferred to LN_2_ dry shippers and transported (with proper permitting) to two animal germplasm repositories for long-term storage (i.e., National Animal Germplasm Program, Fort Collins, CO, USA and Omaha's Henry Doorly Zoo, Omaha, NE). Additionally, although >100 *F. scutaria* sperm samples were successfully cryopreserved (based on post-thaw IVF and viability testing), these cells were not formally banked because this species is not threatened. Likewise, using the basic information generated in this project, we have created a frozen repository for 50 samples of coral embryonic cells (∼10^7^ cells/cryovial) from >60 *F. scutaria* individuals living in Kaneohe Bay that are managed in long-term LN_2_ storage at the Hawaii Institute of Marine Biology.

## Discussion

Little was known historically about coral reproduction until about 30 years ago when massive spawns of multiple species were investigated on the Great Barrier Reef [45], [46], including studies of larvae developmental biology after fertilization *in vitro* [47], [48]. Therefore, it has been evident for decades that an admixture of fresh coral sperm and eggs can robustly produce viable embryos in culture. Our finding of >92% IVF success for fresh *A. palmata* sperm and eggs was consistent with earlier, recent reports for other Acroporids or members of the same family [21], [49]; fertilization success for *F. scutaria*, which has not been studied before in this regard, was slightly lower, but consistently at >75%. Such high values are not normally observed in the ‘IVF world’, especially for traditionally-studied vertebrates (mostly mammals) [50]. Therefore, the consistently high levels of fertilization *in vitro* observed in coral gives the scientific community a valuable metric for examining the impact of a host of basic biological factors that might influence sperm/egg interaction and gamete/embryo form and function (e.g., inter-gamete attractants during spawning events; Morita et al. [51]). In the present study, we determined that IVF was especially useful for assessing the cryosensitivity of coral spermatozoa, helping us determine that significant sperm numbers from two distinctively different species survived freezing stress (to −196°C) and produced developing embryos.

Cryopreservation is a proven method for the long-term maintenance of genetic material for multiple marine species, especially agriculturally important groups, including mollusk sperm, oocytes and embryos [52], [53], [54] and fish sperm (e.g., >200 species, especially salmonids; [43], [55], [56]). The primary justifications for using this technology in these cases have been to maintain gene diversity in distinctive species, subspecies or races, while providing an ‘insurance policy’ in the case of a catastrophe affecting either free-living or *ex situ* populations. One of the most interesting findings of the present work was that corals (at least the two species studied here) defied conventional wisdom for most other previously studied species and biomaterials. Conventional wisdom states that cryosensitivity is highly species- and cell-specific, with differing taxa and cells generally requiring highly explicit freezing and thawing protocols [57], [58]. By contrast, we determined that spermatozoa from *A. palmata* and *F. scutaria* both had high IVF success after cryopreservation as did embryonic cells from *F. scutaria* that expressed post-thaw viability, all from using a simple, 10% solution of DMSO in FSW and a cooling rate of 20 to 30°C/min (sperm) and 0.5°C/min (embryonic cells). These results insinuated that sperm from a diversity of corals may be effectively cryopreserved using a single approach. In fact, these same sperm cryopreservation techniques have been used successfully on two additional Acroporid species (Hagedorn et al, unpublished data). While conclusive experiments on more species are necessary, these initial observations suggested potential broad application to male gametes from Scleractinia (i.e., the stony coral).

Although the basic 10% DMSO and 20 to 30°C/min cooling protocol was effective for spermatozoa from both *A. palmata* and *F. scutaria*, there were some detectable, physiological variations between species, including traits that could ultimately influence field application. Spermatozoa from both species were unaffected by exposure to 5% solutions of DMSO and PG, a concentration considered to be inadequate for safeguarding against intracellular lysis damage during freezing [57], [58]. During our quest to reach the more protective 10% level, we discovered that *A. palmata* sperm motility and fertilization *in vitro* were unaffected by elevated concentrations of both cryoprotectants. By contrast, *F. scutaria* sperm motility and IVF success were highly sensitive to rising cryoprotectant levels. This was important as such a species difference also had practical consequences, specifically limiting the volume of supplemented cryoprotectant (and sperm) to the fertilization vials (to 50 µl). Although this reduced the chance of toxicity, it also meant that the initial sperm concentration had to be very high (10^8^ to 10^9^ cells/ml) to eventually achieve our fertilization target of 10^6^ sperm/ml per vial. While never becoming a significant factor for coral that produce sperm/egg bundles (e.g., *A. palmata*), this could limit success for species that release sperm into the water (e.g., *F. scutaria*). For example, we experienced nights when insufficient amounts of concentrated sperm could be recovered to initiate IVF.

One of our most interesting observations was the night-to-night variability in fertilization success using either fresh or frozen-thawed spermatozoa. Additionally, embryonic cells from *F. scutaria* clearly tolerated cryopreservation better when collected on certain nights than others. However, this variation observed in sperm occurred randomly and was unlinked to any specific night of gamete release and collection. Rather, we suspect that this night-to-night variability indicated that spawned coral spermatozoa (and perhaps eggs) develop fitness over time with the most viable cells being produced nearer to the middle of the spawning period. Alternatively, it may have been possible that the kinetics of spawning were influenced by variations in genotype of our sampled donors.

The night-of-sperm collection had a significant impact on cryosensitivity. From a practical perspective and until more data are generated, it would appear prudent to collect and preserve biomaterials on every night of availability and then test sub-aliquots for viability before culling frozen samples producing poor fertilization results. While the night-of-collection must be considered in the large-scale banking of coral spermatozoa, clearly there is subpopulation of these gametes that is resilient to low temperature exposure and able to survive freezing as demonstrated by viable embryo production. Night-of -collection also influenced sperm motility, which was an issue for *A. palmata*. Ideally, coral sperm samples used for IVF have motility ratings >90%, but some individual colonies of this species had <25% motility values on certain recovery nights. For example, it was our experience that not all *A. palmata* colonies within a given population spawned on the same night or even all in an individual colony. However, over time there was one or two nights when the majority of the colonies spawned, which coincided with the production of those sperm best able to survive the rigors of cryopreservation. By contrast, *F. scutaria*, which spawned over multiple nights throughout the 4 mo summer, consistently produced sperm with both high motility (>90%) and IVF success (>75%). However, after these same sperm were cryopreserved, the incidence of fertilization in culture was highest during July and August and negligible in June or September (data not shown). Therefore, based on observations of dynamic differences in IVF success after imposing a freeze-thaw stress, our findings demonstrated that coral sperm underwent some sort of functional metamorphosis during the overall spawning interval that appeared to enhance fitness and perhaps resilience to stressors.

Here we also reported the first cryopreservation and banking of dissociated coral (*F. scutaria*) embryonic cells. These cells have relevance for ensuring or restoring reef health, for example, testing for and then remediating coral diseases, most of which have gone largely unstudied [13]. To-date, a major obstacle to investigating infectious pathogens within the stony coral has been the inability to maintain primary cultures of differentiated coral tissue for more than a few months [59]–[63]. Frozen embryonic cell lines would offer an inexhaustible resource for large-scale research opportunities, including allowing long-distance transport of specimens to facilitate basic and applied studies. In the long-term future, banked embryonic cells could serve as a resource for stem cells to grow new corals that could be reintroduced into native ecosystems to help sustain or even increase gene diversity.

We envision banked coral sperm eventually being used to help diversify shrinking populations. For example, analyses of *A. palmata* have revealed two genetically-isolated regions within the Caribbean, one in the eastern and one in the western Caribbean with Puerto Rico being a mixed, ‘transition’ zone [64]. Some reef tracts in the west, especially coral stands in the Florida Keys, have little genotypic diversity, with most reefs harboring only one genet (clone) [20]. These genets produce sperm-egg bundles annually, but with no mechanism for self-fertilization and because the distance is too great to neighboring reefs with unrelated genets, reproduction fails to occur [21]. Therefore, these genets living in isolated reef ecosystems likely contribute little to species evolutionary potential. However, if collected and stored sperm were available from these various isolates, then opportunities for introducing new genes (and even increasing gene diversity) would be possible, all of course under the guidelines of an appropriate and official conservation plan. Our findings here that frozen coral sperm can be used to produce embryos mean that this option could likely be feasible for federal and state agencies and NGOs charged with the formidable challenges of preserving marine resources. The development of genome resource banks containing coral sperm and embryonic cells would: 1) preserve all existing gene diversity if not the species themselves, especially those that are under high risk of extirpation or extinction; 2) store the entire genome, including as yet unknown but critically valuable epigenetic factors; 3) create opportunities for diversifying shrinking populations by avoiding natural losses in heterozygosity due to genetic drift; and 4) produce substantial amounts of scholarly knowledge on these invertebrate taxa that have been far understudied in the physiological/reproductive sciences. Of course, major hurdles remain, especially developing tools and protocols to achieve consistent settlement, recruitment and growth of developing coral. Regardless, the financial expenses of applying these simple cryopreservation protocols are quite small compared to the costs of potential ecosystem-wide losses. This concept appears especially timely given the many growing local and global stressors imposed on coral reefs [4], [5], [6].
